# Effects of Physical Activity on Inhibitory Function in Children with Attention Deficit Hyperactivity Disorder: A Systematic Review and Meta-Analysis

**DOI:** 10.3390/ijerph20021032

**Published:** 2023-01-06

**Authors:** Meng Wang, Xinyue Yang, Jing Yu, Jian Zhu, Hyun-Duck Kim, Angelita Cruz

**Affiliations:** 1 Department of Physical Education, Keimyung University, Daegu 42601, Republic of Korea; 2 College of Sports Science, Shenyang Normal University, Shenyang 110034, China; 3 Department of Physical Education, Zhongyuan University of Technology, Zhengzhou 450007, China; 4 Department of Sport Marketing, Keimyung University, Daegu 42601, Republic of Korea

**Keywords:** ADHD, children, cognition, executive function, physical activity dose

## Abstract

This systematic review and meta-analysis aimed to systematically evaluate the effect of physical activity (PA) on inhibitory function in children with ADHD. Experimental studies on the effect of PA on the inhibitory function of children with ADHD were retrieved. The data were obtained from PubMed, The Cochrane Library, Web of Science, EBSCO (MEDLINE, APA Psyclnfo, ERIC), Embase, Scopus, and ProQuest. The search period was from the date of inception of the respective databases to 4 May 2022, and Reviewer Manager software (version 5.3) was used for analysis. Eleven articles and 713 samples were included in the meta-analysis. Results revealed that PA can significantly improve the inhibitory function of children with ADHD (SMD = 0.78, 95% CI: 0.45–1.10, *p* < 0.001). Subgroup analysis showed that the effectiveness of PA as an intervention in improving the inhibitory function of children with ADHD was moderated by the frequency, intensity, duration, type, and length of intervention. Based on the findings, PA can effectively improve interference suppression inhibitory function in children with ADHD. Longitudinal open-skill exercise for 60 min or more, two times/week has the best effect on improving inhibitory function in children with ADHD.

## 1. Introduction

Attention deficit hyperactivity disorder (ADHD) is the most common neurodevelopmental disorder in children, with a global prevalence of approximately 5.2–7.2% [1]. Although considered to be a childhood disease, it is reported that in most cases (approximately 65%), symptoms persist in adulthood [2]. ADHD can result in poor educational performance, job unemployment, marital failure, crimes [3,4], and various mental illness, including personality disorder, self-harm, affective disorder, and drug abuse [5,6,7]. An important defect of ADHD is inhibition dysfunction disorder [8,9]. Inhibition function is to control one’s attention, thought, emotions and behavior, and to overcome strong external temptations or internal tendencies. Inhibition function also enables people to focus, resist external interference, and become more focused [10]. Presently, multiple hypotheses of the pathogenesis of ADHD exist, but among the cognitive psychology theories, the most influential and widely accepted is Barkley’s response inhibition model theory [11], which argues that response inhibition defects in ADHD lead to secondary impairment of executive function, resulting in symptoms such as attention deficit, hyperactivity, and impulsivity. Impairment of inhibitory function is the primary cause of ADHD symptoms [11,12]. Inhibitory function defects in children with ADHD lead to problems such as personality disorders, self-harm, and antisocial behavior [13]. Improvement of inhibitory function plays an important role in regulating children’s academic achievement, self-regulation, and social emotion [14,15,16,17]. Therefore, interventional therapy—consisting of medication, behavioral, and psychological interventions—aimed at treating inhibitory function is necessary for children’s future development [18,19]. Therapeutic drugs have been in common use, but their effect on inhibitory function is not significant [20], and approximately 30% of patients suffered adverse reactions or drug tolerance [21,22]. Behavioral and psychological interventions, on the contrary, can be cumbersome for parents and children, and the treatment is difficult to sustain [18,19,23,24,25].

Recently, physical activity (PA) has gained increasing attention from researchers as a novel non-pharmacological intervention approach to managing children with ADHD, particularly their inhibitory function [26]. Physical activity refers to any physical activity that causes energy consumption due to skeletal muscle contraction, including daily work, housework, physical exercise, and entertainment activities [27]. Physical exercise and sports are often regarded as the inferior concept of physical activity [28]. Evidence suggests that PA can influence children’s inhibitory function. However, the findings differ in the dose and outcome of intervention among studies, raising confusion about its effectiveness and applicability. For instance, some research results pointed out that football training for 35 min each time, two times/week, for a total of eight weeks [29] and running training for 30 min each time can improve the inhibitory function of ADHD children [29]; whereas another study showed that aerobic running for 30 min each time did not significantly improve inhibition function [30].

Likewise, there is a lack of clarity on the PA duration among study reports; some indicate that long-term intervention can improve the inhibitory function of ADHD children [30], while others reveal that short-term intervention can improve it significantly [31]. Additionally, some studies show that moderate-intensity aerobic exercise has a positive effect on children’s inhibitory function [29,32,33], whereas others indicate that high-intensity exercise has a significant effect [34,35]. Certain studies have pointed out that open-skill intervention has more advantages than closed-skill [36,37]. Studies also differed on the PA frequency: once [31], twice [29], or thrice per week [38]. Considering the uncertainty of the effect of PA on the inhibitory function of ADHD and the debate on the dose of PA, it is necessary to systematically summarize the effect of PA on the inhibitory function of ADHD children and the appropriate dose of PA for children, so as to provide a theoretical basis for subsequent studies.

Consequently, the main purpose of this review is to systematically summarize the effect of PA on the inhibitory function of children with ADHD, with a particular focus on identifying the appropriate dose of PA for children with ADHD. We also examined sources of heterogeneity that affected variations between studies in relation to PA interventions.

## 2. Research Method

According to the requirements of the Preferred Reporting Items for Systematic Reviews and Meta-Analyses (PRISMA) [39], data collation and statistical analysis were performed for the included studies.

### 2.1. Literature Retrieval Strategy

The following bibliographic databases were searched without restriction on language or publication year or publication type: PubMed, The Cochrane Library, Web of Science, EBSCO (MEDLINE, APA Psyclnfo, ERIC), Embase, Scopus, and ProQuest, from inception to 5 April 2022.

Furthermore, the reference lists of included studies and systematic reviews from the last 10 years on the effect of PA on inhibitory function in children with ADHD were further scrutinized. The search keywords included “attention deficit disorders with hyperactivity”, “PA”, “children”, “inhibition”, and “psychological”. Retrieval combines keywords and free words, which are determined by repeated pre-checks and supplemented by manual retrieval of gray literature.

After literature retrieval, two researchers screened it by adopting the independent double-blind method. First, the literature was imported into Endnote X9 software (Clavirate, Philadelphia, PA, USA) for deduplication, and then the titles and abstracts were read to preliminarily screen the articles. The full texts of the studies that might be included were downloaded, and the full texts were read meticulously. After screening, two researchers compared the extracted literature; conflicts, if any, were resolved with the help of a third researcher through a joint discussion.

### 2.2. Inclusion Criteria

The following studies were included in this systematic review and meta-analysis: (1) Studies that included children with ADHD who conformed to the diagnostic criteria in the Diagnostic and Statistical Manual of Mental Disorders (DSM-4, DSM-5) or the tenth revision of the International Statistical Classification of Diseases and Health-related Problems (ICD-10); (2) Studies that investigated the effect of exercise on inhibitory function of ADHD, with the independent variable as “PA” (exercise, etc.) and the dependent variable as “the effect of PA on the inhibitory function of children”; (3) Studies that obtained the mean and standard deviation of the inhibitory function of the intervention group and control groups directly or indirectly; (4) Studies that were designed as a randomized controlled trial; (5) Studies that reported no significant differences between the intervention and control groups before the experiment; (6) Studies that included participants of <18 years of age.

### 2.3. Exclusion Criteria

The following studies were excluded from this systematic review and meta-analysis: (1) Studies that included participants that were diagnosed with dementia and mental health disorders; (2) Studies that did not have a control group; (3) Studies that had an intervention program containing confounding factors of non-exercise intervention, such as cognitive training, vitamin supplements, and drug intervention; (4) Studies with duplicate publications or poor quality assessments; (5) Reviews, surveys, and conference or case papers.

### 2.4. Data Extraction and Coding

Two researchers independently extracted the literature, extracted relevant information using a pre-designed table, and coded it. Extraction and coding contents included: (1) author, nationality, and publication year; (2) intervention duration, frequency, period, exercise intensity, sample size, and study design; and (3) outcome indicators: the effect of intervention (PA) on children’s inhibitory function after a variety of tasks. Indicators were extracted in the form of means and standard deviations. If a paper included more than one set of data, it was counted as multiple studies.

### 2.5. Literature Screening and Quality Evaluation

Conducted by 2 researchers using the Cochrane risk assessment tool, the evaluation indicators included seven items: random sequence generation (selection bias), allocation concealment (selection bias), blinding of participants and personnel (performance bias), blinding of outcome assessment (detection bias), incomplete outcome data (attrition bias), and selective reporting (reporting bias). Based on the evaluation criteria, the literature was divided into three categories: low risk of bias, high risk of bias, and unclear risk of bias (lack of relevant information or uncertainty of bias).

### 2.6. Statistical Method

The Review Manager Software version 5.3 (RevMan 5.3; Copenhagen, Denmark: The Nordic Cochrane Centre, The Cochrane Collaboration) was used to perform the analyses for the combined effect, sensitivity, and subgroups. The combined effect was calculated as mean ± standard deviation, and 0.2, 0.5, and 0.8 were determined to be the thresholds for small, medium, and large effect sizes [40], respectively. Heterogeneity was quantified by the I^2^ statistic [41], and 25%, 50%, and 75% were determined to be low, medium, and high ratios of interstudy heterogeneity, respectively. Sensitivity analysis used a changing statistical model and literature elimination method to compare the changes in standardized mean difference (SMD), 95% confidence interval (CI), and I^2^ between the two groups and to test the stability of the analysis results. Begg’s test with STATA 15.0 software (StataCorp LLC, Cambridge, UK) was used to test publication bias.

## 3. Results

### 3.1. Literature Retrieval

**Figure 1:**
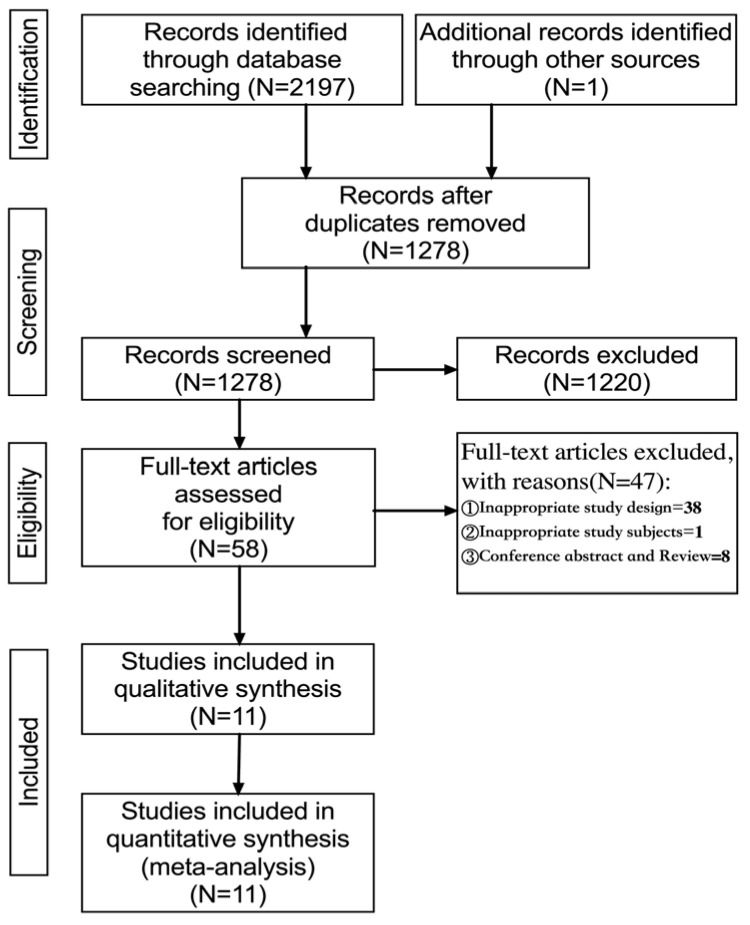
Flowchart of the study selection process.

A total of 2198 articles were identified by searching PubMed (*n* = 171), The Cochrane Library (*n* = 139), Web of Science (*n* = 731), EBSCO (MEDLINE, APA Psyclnfo, ERIC) (*n* = 150), Embase (*n* = 184), Scopus (*n* = 526), ProQuest (*n* = 296), and one article was manually identified. The articles were then imported into Endnote X9, and 1278 were obtained after deduplication. Sixty-two articles were initially screened by reading their titles and abstracts, and finally, 11 were selected for the quantitative synthesis (meta-analysis) (Figure 1).

### 3.2. Characteristics and Quality Evaluation of the Included Literature

A preliminary search was conducted with 2198 articles and 11 articles were selected. The study designs of included studies were as follows: randomized controlled (*n* = 8), crossover (*n* = 1), and parallel group (*n* = 2) trials. Included studies had 713 participants in total: 362 in the experimental group and 351 in the control group. The total number of participants in each study ranged from 14–120. The included studies (Table 1) reported PA variables, including table tennis, aerobic dance, walking, and somatosensory games but not all of them reported exercise intensity; the PA duration ranged from 15–60 min/day, with a frequency of 1–3 times/day, for 8–12 weeks.

**Figure 2:**
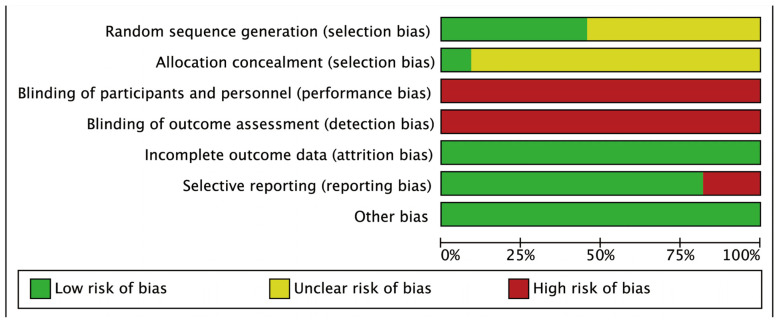
Diagram for the risk of bias.

The Cochrane systematic evaluation criteria were used to comprehensively evaluate the risk of bias in the included articles. As shown in Figure 2, the 11 studies had certain biases, because, primarily, it is difficult to conduct double-blind trials in PA intervention experiments (only one study clearly stated that a single-blind design was used) [42].

### 3.3. Results of Meta-Analysis

**Figure 3:**
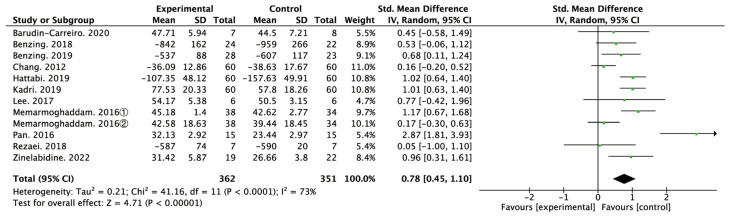
Forest plot of meta-analysis of PA on inhibition [29,38,42,43,44,45,46,47,48,49,50].

According to the forest plot (Figure 3) for the effect of PA intervention on inhibitory function in the 11 studies, the heterogeneity (I^2^) was 73% (>50%) and for the Q test, the *p* value was 0.00 (<0.05), suggesting that there was moderate heterogeneity among the selected studies in this study; random effects were selected for meta-analysis. The results of the meta-analysis based on random effects showed that the heterogeneity (SMD = 0.78, 95% CI: 0.45–1.10, *p* = 0.00) and Begg’s test results (Z = 4.78, *p* = 0.00) were statistically significant, indicating that PA effectively improved the inhibitory function of children with ADHD (Figure 3).

To explore whether heterogeneity among the studies was owing to a single study, a sensitivity analysis was carried out by eliminating them one at a time. The result showed that SMD ranges from 0.66–0.85, and the range of I^2^ is 60–76%, respectively (*p* < 0.05 each), indicating low sensitivity of data and certain stability and reliability of the results.

To further explore the source of potential heterogeneity, subgroup analysis of potential moderators was conducted (Table 2).

The results of subgroup analysis through subgroup types are shown in Table 3. According to subgroup analysis, results from the combined six studies [38,44,46,47,49] in the three times/week PA frequency group showed that there was moderate heterogeneity among the studies within the group. The combined effect size was calculated using the random effects model, and the difference was statistically significant, indicating that a PA frequency of three times/week could effectively improve the inhibitory function of children with ADHD.

Results from the combined three studies [31,45,48] in the two times/week PA frequency group showed a high degree of heterogeneity among the studies within the group. The random effects model was used to calculate the combined effect size, and the difference was statistically significant, indicating that a PA frequency of two times/week effectively improved the inhibitory function of children with ADHD.

Results from the combined three studies [29,42,43] in the one time/week PA frequency group showed that there was neither heterogeneity among studies within the group nor a statistically significant difference in the combined effect size using the fixed effect model. This indicates that PA one time/week cannot effectively improve the inhibitory function of children with ADHD.

Interestingly, from the perspective of effect size, a PA frequency of two times/week had a more positive intervention effect than that of three times/week.

Subgroup analysis was also conducted according to the session time. Present review findings revealed that results from the combined three studies [29,42,43] in the PA with the short-interval exercises group showed that there was no heterogeneity among studies within the group. The fixed-effect model was used to calculate the combined effect size, and the difference was not statistically significant, indicating that short-interval PA could not effectively improve children’s inhibitory function. The findings from the combined four studies [44,45,49,50] in the moderate-interval PA group showed low heterogeneity among studies within the group. The fixed-effect model was used to calculate the combined effect size, and the difference was statistically significant, indicating that moderate-interval PA effectively improved the inhibitory function of children with ADHD. The findings from the combined five studies [38,46,47,48] in the long-interval PA group showed high heterogeneity among the studies within the group. Random effects model was used to calculate the combined effect size, and the difference was statistically significant, indicating that long-duration PA effectively improved the inhibitory function of children. Additionally, in terms of the amount of effect, long-interval PA had a more positive effect than moderate-interval PA.

Subgroup analysis was also performed according to the duration of the intervention. The acute intervention findings from the combined three studies [29,42,43] showed no heterogeneity among the studies within the group. The fixed-effect model was used to calculate the combined effect size, and the difference was not statistically significant, indicating that a single intervention of PA did not improve the inhibitory function of children with ADHD. The results from the combined nine longitudinal studies [38,44,45,46,47,48,49,50] showed a high degree of heterogeneity among studies within the group, and the combined effect size was statistically significant using the random effects model, suggesting that the long-term PA intervention effectively improved the inhibitory function of children with ADHD.

Subgroup analyses were additionally conducted based on the type of PA. The results from the combined four studies [29,42,49,50] that used closed-skill sports showed low inter-study heterogeneity within groups. The fixed-effect model was used to calculate the combined effect size, and the difference was statistically significant, indicating that closed-skill sports can effectively improve the inhibitory function of children with ADHD. The results of open-skill sports from the combined four studies [45,47,48] showed high heterogeneity among the studies within the group. The random effects model was used to calculate the combined effect size, and the difference was statistically significant, indicating that open-skill sports can effectively improve the inhibitory function of children with ADHD.

Results from the combined two studies [38,46] of motor skill training and results from the combined two studies [43,44] of exergaming both showed no heterogeneity among studies within the group. The fixed effects model was used to calculate the combined effect size, and the difference was statistically significant, indicating that both motor skill training and exergaming can effectively improve the inhibitory function of children with ADHD. Open-skill sports, motor skill training, exergaming, and closed-skill sports significantly improved the inhibitory function of children with ADHD. However, open-skill sports, motor skill training, and exergaming had more positive intervention effects than closed-skill sports.

The result from the combined four studies [43,46,47,50] of the MVPA showed that there was moderate heterogeneity among the studies within the group. The random effect model was used to calculate the combined effect, and the difference was statistically significant, indicating that moderate to high intensity can effectively improve the inhibitory function of children. The result from the combined two studies [29,38] of the MPA showed that there was a high degree of heterogeneity among the studies within the group. The combined effects were calculated by the random effect model, and the difference was not statistically significant.

The result from the combined 10 studies [29,38,42,43,44,45,46,47,48,49,50] of the interference suppression group showed that there was high heterogeneity among the studies within the group. The random effect model was used to calculate the combined effect, and the difference was statistically significant, indicating that after PA intervention, the performance and ability of interference suppression were significantly improved. The result from the combined two studies [44,47] of the response inhibition group showed that there was moderate heterogeneity among the studies. The random effect model was used to combine the effect amount, and the difference was not statistically significant.

**Figure 4:**
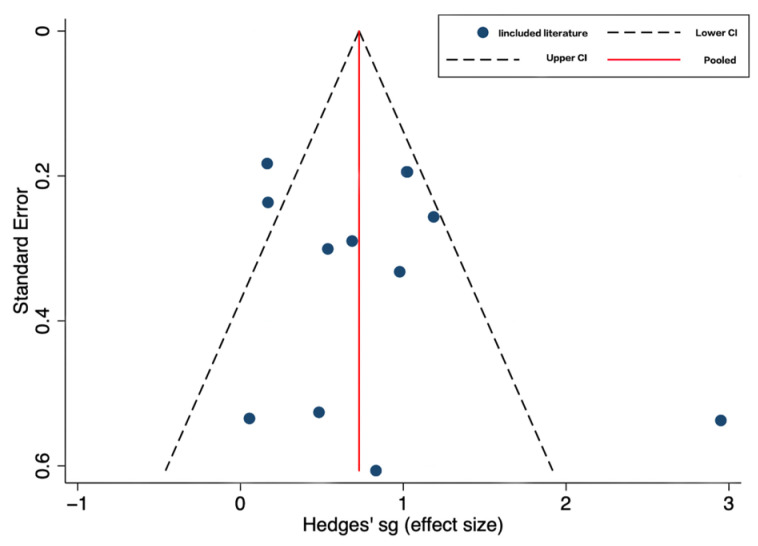
Funnel plot.

The funnel chart (Figure 4) shows that the figure is symmetrical, and the Begg’s test results show that Z = 0.48 *p* > |z| = 0.631, indicating that there was no publication bias in this study.

## 4. Discussion

This study aimed to examine the effect of PA on the inhibitory function of children with ADHD. Quantitative analysis of 11 relevant studies showed that the effect of PA on the inhibitory function of children was 0.78 (95% CI: 0.45–1.10). According to the effect evaluation standard proposed by Cohen, PA had a moderate-to-large significant effect on the inhibitory function in children with ADHD. This result confirms the previous meta-analyses findings about the facilitatory effect of PA not only on general cognition but also on inhibition in children with ADHD [46,56]. Subsequent analysis showed that frequency, intensity, duration, type, and length of intervention significantly moderated the effect of PA in facilitating inhibition control in children with ADHD.

### 4.1. Type of PA

Studies have shown that aerobic exercise training can bring functional plastic changes in brain areas such as the left inferior frontal gyrus [46,56], and previous meta-analyses results indicated that aerobic exercise has a positive impact on the inhibition control of children with ADHD [29,57,58,59]. While present study findings corroborate those of these previous reports [57,58,59], the finding that type of exercise did not moderate the effect of PA on inhibitory control of children with ADHD contradicts previous reports. This disparity in results may be explained by the differences in the included studies in the meta-analyses. Liang et al. [59]. and Tan et al. [60]. evaluated studies that included the three core domains of cognition, whereas only the inhibitory aspect of the executive function was investigated in the present study.

Closed-skill sports (rhythmic gymnastics, swimming, and classical ballet) improve the inhibitory function of children with ADHD. However, a more positive impact on inhibition function with open-skill sports (football, basketball, volleyball, and martial arts) than with closed-skill sports training has been reported [36,61,62]. A study demonstrated that closed-skill group training had no inhibitory control advantage, compared with open-skill group training [63]. The present review findings are consistent with the aforementioned results. In this review, two included studies implemented motor skill training (which involved ball and rope jumping and water aerobic exercise). A combination of aerobic training and skill training is more effective than aerobic training alone in improving inhibitory function [64] and combination training (rope skipping and ball games) has a positive impact on executive function [46]. The results are consistent with our findings, indicating that motor skill training has a more positive intervention effect on the inhibitory function of ADHD than closed-skill sports.

Two studies implemented exergaming interventions (exercise gaming with motion sensing games), which combine cognitive activity with PA and can stimulate increased PA while gaming [65]. Some studies have shown that motion-sensing games can promote neuro-remodeling effects, improving certain cognitive functions in the brain [66]. Moreover, studies have found that motion-sensing games with high-intensity PA can promote children’s inhibitory function [67]. Present review findings are consistent with these findings. The motion-sensing games had a significant effect on the inhibitory function of children with ADHD and a more positive effect compared with closed-skill sports. This may be attributed to the fact that participants need to adapt to the constantly changing environment; therefore, there are more cognitive requirements for visual-spatial ability and inhibitory function [53,63,68].

Closed-skill sports are, however, performed in a predictable and stable environment where participants do not need to be stimulated by multiple senses [53,69]. It is inferred that children with ADHD tend to give priority to the use of visual and auditory receptors in open-skill sports, motor skill training, and exergaming training, while they tend to use proprioceptors in closed-skill sports, which prioritizes the training of receptors and their dominant neural mechanisms in subsequent training. For example, studies have pointed out that children with ADHD also have impaired visuospatial attention ability [8]; the right parietal lobe, which may control bilateral visuospatial attention can be activated by PA during cognitive activities performed in ADHD disorder [70,71], indicating that PA can improve children’s visuospatial attention problems. Some studies have concluded that open-skill sports are better than closed-skill sports [61,62] in enhancing audiovisual perception after training. This is consistent with the results of the present study. Although the existing evidence tends to support that open-skill sports are more effective in improving cognitive ability (i.e., visual-spatial attention, inhibition, etc.) than closed-skill sports, the underlying mechanism is still unclear and needs further exploration.

### 4.2. Length of Intervention

In the present review, long-term intervention was found to have a significant effect on the inhibitory function of children with ADHD, whereas acute PA had no significant effect. A previous meta-analysis showed that longitudinal PA has the effect of improving inhibitory function, whereas the effect of acute PA intervention is only temporary [59]. Studies have suggested that longitudinal PA may enhance neural connections [72]. A longer period of PA can produce better cognitive changes [73], raise the levels of brain-derived neurotrophic factor (BDNF) [74], and enhance the release of dopamine [75]. Relevant meta-analysis also suggested that longitudinal sports activities may promote nerve growth and development, and the impact may exist for a long time. Regular sports activities can more effectively improve children’s ADHD symptoms [76]. Moreover, a recent meta-analysis indicated that longitudinal PA intervention had a medium to large significant impact [58,60]. The above conclusions are consistent with this study. Therefore, long-term PA plays an important role and is more conducive to the improvement of inhibitory function in children with ADHD.

### 4.3. Duration of PA

In terms of session duration, we found that while both moderate- and long-duration PA could significantly affect the inhibition of ADHD in children, the effect of long-term PA was far greater than that of moderate-duration PA. The results suggest that a PA duration of at least 45 min, or better yet, longer than 60 min per session can greatly improve the inhibitory function of children with ADHD. Interestingly, this finding contradicts the meta-analysis results that showed a lack of significant moderating effect of exercise duration between PA and executive functions in children with ADHD [60,61]. The disparity in results may be due to the inclusion of studies that examined various executive functions in children with ADHD as outcome variables, while the current review only focused on a specific component of cognition. Further, a prior meta-analysis revealed that short-duration (15–30 min) exercise was already substantial in enhancing the inhibitory control of children with ADHD [77], however, this contradicts our review findings that training less than 45 min had no beneficial effect on inhibition. The type of intervention and study inclusions may be potential reasons for the differences in results, wherein the previous meta-analysis only examined the acute effect of PA on inhibition, whereas the current study considered both acute and long-term impacts of PA.

On the contrary, the present results not only support previous studies that 40–60 min of PA each time can improve the inhibitory function of children [78,79], but also adds to the literature by demonstrating that longer duration PA each time can provide even greater benefits for children with ADHD. However, further studies are needed to determine the optimal session time.

### 4.4. Frequency of PA

Present review findings revealed that, while both two and three times/week of PA can significantly enhance the inhibitory function of children with ADHD, interestingly, PA two times/week has a greater effect on the inhibitory function of children than PA three times/week. This outcome may be due to the characteristics of children with ADHD, who are often physically inactive or overweight/obese. A study showed that children with ADHD did not engage in regular PA [46]. Lower amounts of PA can lead to physical fatigue. Fatigue has a negative impact on cognitive performance [80], resulting in unsatisfactory intervention outcomes.

In addition, studies have shown that there is a significant relationship between ADHD and obesity or overweight [81], wherein the defect in inhibition function strengthens the irregular diet of patients [82], causing obesity or overweight. The influence of PA on cognitive ability is regulated by many factors [60], if intervention training is carried out in children with ADHD who lack exercise and/or obesity, they are likely to experience unpleasant feelings and physical discomfort with a high PA dose, resulting in the rejection of intervention training. Further analysis of studies that examined PA two times/week indicated that the level of physical function of children with ADHD improved and the degree of completion of intervention was higher in the subsequent stages, thus producing more significant effects. However, more studies are needed to further explore the optimal number of weekly physical activities.

### 4.5. Intensity of PA

In terms of the intensity of PA, the present review findings state that moderate-to-high-intensity exercise had a large effect on the inhibitory function of children with ADHD. Although this result confirms a previous meta-analysis that found that moderate to vigorous PA had a significant positive (small) effect on executive function in children with ADHD, it only investigated the effects of acute bouts of PA on executive functions in children with ADHD. Thus, the current review adds to the literature by demonstrating that longer interventions of moderate to vigorous PA can provide even larger effects on higher-order executive function, particularly inhibitory function in children with ADHD.

In contrast, this finding differs from a previous meta-analysis that found that moderate-intensity PA alone has a moderate-to-large effect on inhibitory function in children with ADHD compared with light, moderate to vigorous, and vigorous physical activities [59]. The inclusion of eligible studies could be a potential reason for such disparity in outcomes with more recent studies and more participants in the present study than in prior reviews.

However, further examination is essential to identify the appropriate intensity level of PA to be provided and maximize the benefits of PA in enhancing inhibitory control.

### 4.6. Inhibitory Function Types and PA

An important defect of ADHD is inhibition dysfunction disorder [8,9]. Harnishfeger et al. classified inhibition function into interference suppression and response inhibition [83,84]. Response inhibition refers to the ability to inhibit responses that do not meet current needs or inappropriate behaviors. Interference suppression can prevent interference information from affecting working memory, thus improving the efficiency of working memory processing useful information. The interference suppression tests for ADHD children included in this study include the Stroop task [29,38,42,45,46,47,48,50], CPT [49], and Flanker task [43]; the other two papers used the Simon task [44] and Go/No-go task [47] to perform response inhibition tests on children with ADHD. The obvious problem of ADHD children is that they find it difficult to concentrate and are easily interfered with by irrelevant information [85]. Studies have pointed out that ADHD children have interference suppression [86] and response inhibition defects [87]. This study concluded that PA intervention can effectively improve the interference suppression ability, which plays an important role in improving the symptoms of children with ADHD.

The World Health Organization’s (WHO) guidelines on PA and sedentary behavior (2020) [88] suggest that to obtain the general health benefits of PA in addition to enhancing cognition/ reducing the risk of cognitive impairment, children and adolescents living with disability (which includes individuals with ADHD) are recommended to be provided with opportunities or encouragement to perform at least an average of 60 min/day of moderate-to-vigorous aerobic PA across the week. Vigorous-intensity aerobic physical activities and muscle- and bone-strengthening activities at least three times/week are also strongly recommended. Present review findings regarding the appropriate PA dose to facilitate inhibitory control in children with ADHD lend credibility to the majority of the PA guidelines, as suggested by WHO experts. Contrarily, it is still uncertain how bone-strengthening activities, such as resistance training, can affect inhibitory control in children with ADHD, which, therefore, needs further examination.

## 5. Limitations

The study has some limitations. The reliability of the results in this review may be affected by confounding factors such as the non-standardization of PA variables in the included studies (frequency, session time, length, and type), their inclusion criteria, and their patient compliance. First, there were methodological defects in the included studies. A few randomized controlled trials were included in this study. Most studies failed to use the blind method and included only published literature. Second, as different studies have focused on different subcomponents of inhibitory function, the present review could not analyze all the subcomponents owing to the disparity in the quality of the included literature. Third, there was a lack of unified standards for PA intensity in the included literature, and only some studies defined the level of PA intensity. Thus, no subgroup analysis for moderate PA was conducted due to insufficient data. More literature needs to be included in the response inhibition group to verify the results. Finally, owing to the small number of included studies in this review, further subgroup analysis could not be performed and the status of medication could not be evaluated.

## 6. Conclusions

PA can effectively improve the inhibitory function of children with ADHD, and the effect of PA interventions needs to be verified by more empirical studies. Long-term moderate-to-vigorous open-skill exercise, two times/week, 60 min or more each time had the best effect in improving the interference suppression of the inhibitory function of children with ADHD. In the future, a randomized controlled study design should be used to improve the quality of the methodology in exploring the specific effect of PA on inhibitory function in children with ADHD.

## Figures and Tables

**Table 1:** Basic characteristics of the literature included in the study.

Study	Country	Diagnostic Criteria	PA Content	Research Design	Experimental Group			Control Group		End Indicator
					Session Time; Frequency; Period	Sample	Exercise Intensity	Sample	Activity Mode	
Barudin-Carreiro 2020 [42]	America	DSM-IV	walk	RCT	20 min/1 time	7	NA	8	sit	Stroop Test
Benzing 2018 [43]	Switzerland	ICD-10	Body feeling game	RCT	15 min/1 time	24	MVPA	22	watch video	Flanker Task
Benzing 2019 [44]	Switzerland	ICD-10	Body feeling game	parallel group	30 min/week 3 times/8 weeks	28	NA	23	Non Exercise	Simon Task
Chang 2012 [29]	Taiwan, China	DSM-IV	Run	RCT	30 min/1 time	20	MPA	20	watch video	Stroop Test
Hattabi 2019 [38]	Tunisia	DSM-IV	water aerobic exercise	RCT	90 min/week 3 times/12 weeks	20	MPA	20	Non Exercise	Stroop Test
Kadri 2019 [45]	Tunisia	NA	Taekwondo	RCT	50 min/week 2 times/64 weeks	20	NA	20	Non Exercise	Stroop Test
Lee 2017 [46]	South Korea	DSM-IV	Ball; Rope-jumping	RCT	60 min /week 3 times/12 weeks	6	MVPA	6	Non Exercise	Stroop Test
Memarmoghaddam 2016 [47]	Iran	DSM-IV	pingpong	RCT	90 min/week 3 times/8 weeks	19	MVPA	17	Non Exercise	Stroop Test; Go-No Go Task
Pan 2016 [48]	Taiwan, China	DSM-IV	pingpong	Crossover	70 min/week 2 times/12 weeks	15	NA	15	Non Exercise	Stroop Test
Rezaei 2018 [49]	Iran	DSM-5	Yoga	parallel group	45 min/week 3 times/8 weeks	7	NA	7	Non Exercise	CPT
Zinelabidine 2022 [50]	Tunisia	NA	Aerobic Dance	RCT	45 min /week 2 times/8 weeks	19	MVPA	22	Non Exercise	Stroop Test

Note: DSM-IV: Diagnostic and Statistical Manual of Mental Disorders, 4th edition; DSM-5: Diagnostic and Statistical Manual of Mental Disorders, 5th edition; ICD-10: the International Statistical Classification of Diseases and Health-related Problems. 10th edition; CPT: continuous operation test; NA: no data is provided; RCT: randomized controlled trial; MPA: moderate physical activity; MVPA: moderate to vigorous physical activity; PA: physical activity.

**Table 2:** Selection of intervention characteristics as moderators.

Moderator	Level	Hypothesis
**Frequency of PA**	(a) One time per week; (b) 2 times per week; (c) 3 times per week.	It is hypothesized that PA more than twice per week for children with ADHD leads to benign inhibitory function (code has previously been used) [51].
**Session time**	(a) Short (<45 min); (b) Moderate (45–60 min); (c) Long (≥60 min).	We hypothesized that long-interval (≥60 min) PA intervention for children with ADHD produced more effects than moderate or short one (code previously used) [52].
**Length of intervention**	(a) Acute; (b) Longitudinal.	(a) Acute PA within 10 to 60 min; (b) PA that was provided over several weeks in a long-term intervention plan. It was hypothesized that long-term intervention would lead to greater benign changes in inhibitory function for ADHD children than acute intervention (code previously used) [52].
**Type of PA**	(a) Closed-skill sport (Aerobic exercise); (b) Open-skill sport (e.g., pingpong, Taekwondo); (c) Motor skill training; (d) Exergaming.	Closed-skill sport is defined as a sport in which the sport environment is relatively highly consistent, predictable, and self-controlled like running or swimming [53,54]. Open-skill sport is defined as a sport that requires reaction in a complex environment with dynamic changes, and unpredictable and uncontrollable rhythms (e.g., ping-pong, taekwondo) [54]. It is assumed that open-skill sports can improve children’s inhibitory function more effectively than other physical activities (code has been used before) [55].
**Intensity of PA**	(a) Moderate to vigorous physical activity (MVPA); (b) Moderate physical activity (MPA).	We hypothesized that MVPA PA intervention in ADHD children would produce a larger effect than MPA.
**Inhibition type**	(a) Interference suppression; (b) Response inhibition.	The measurement of inhibition function is divided into two dimensions: response inhibition ability measurement and interference suppression ability measurement. The research paradigms of response inhibition mainly include Go/No-go tasks, stop-signal tasks, etc. The research paradigms of interference mainly include the Stroop task, Flanker task, etc [10]. The inhibition function is divided according to the research paradigms included in the article. We hypothesized that PA intervention would have a significant impact on interference suppression and response inhibition in children with ADHD.

Note: PA, physical activity; ADHD, attention deficit and hyperactivity disorder.

**Table 3:** Subgroup analysis of the effect of physical activity variables on inhibition.

Variable	Grouping Criteria	n	Heterogeneity Test Result			Effects Model	Effect Size Test		
			Q	P	I^2^/%		ES (95% CI)	z	P
**Frequency of PA**	one time per week	3	1.19	0.55	0	Fixed	0.28 (−0.02, 0.57)	1.85	0.06
	two times per week	3	10.97	0	82	Random	1.49 (0.60, 2.37)	3.29	0
	three times per week	6	12.48	0.03	60	Random	0.70 (0.31, 1.08)	3.56	0
**Session time**	Short (<45 min)	3	1.19	0.55	0	Fixed	0.28 (−0.02, 0.57)	1.85	0.06
	Moderate (45–60 min)	4	3.41	0.33	12	Fixed	0.86 (0.58, 1.13)	6.13	0
	Long (≥60 min)	5	24.48	0	84	Random	1.11 (0.45, 1.78)	3.27	0
**Length of intervention**	Acute	3	1.19	0.55	0	Fixed	0.28 (−0.02, 0.57)	1.85	0.06
	Longitudinal	9	27.96	0	71	Random	0.92 (0.55, 1.29)	4.89	0
**Type of PA**	closed-skill sport	4	4.74	0.19	37	Fixed	0.33 (0.05, 0.62)	2.27	0.02
	open-skill sport	4	24.4	0	88	Random	1.18 (0.42, 1.94)	3.05	0
	Motor skill training	2	0.15	0.69	0	Fixed	1.00 (0.63, 1.36)	5.38	0
	Exergaming	2	0.13	0.72	0	Fixed	0.60 (0.20, 1.01)	2.9	0
**Intensity of PA**	Moderate to vigorous physical activity (MVPA)	4	9.38	0.05	57	Random	0.70 (0.28, 1.12)	3.24	0
	Moderate physical activity (MPA)	2	10.28	0	90	Random	0.59 (−0.25, 1.43)	1.38	0.17
**Inhibition type**	Interference suppression	10	34.89	0	74	Random	0.78 (0.45, 1.10)	4.57	0
	Response inhibition	2	1.84	0.17	46	Random	0.39 (−0.10, 0.89)	1.56	0.12

## Data Availability

Not applicable.
